# Mobile-surface bubbles and droplets coalesce faster but bounce stronger

**DOI:** 10.1126/sciadv.aaw4292

**Published:** 2019-10-25

**Authors:** Ivan U. Vakarelski, Fan Yang, Yuan Si Tian, Er Qiang Li, Derek Y. C. Chan, Sigurdur T. Thoroddsen

**Affiliations:** 1 Division of Physical Science and Engineering, King Abdullah University of Science and Technology (KAUST), Thuwal 23955-6900, Saudi Arabia.; 2 Department of Modern Mechanics, University of Science and Technology of China, Hefei 230027, China.; 3 School of Mathematics and Statistics, University of Melbourne, Parkville, VIC 3010, Australia.; 4 Department of Mathematics, Swinburne University of Technology, Hawthorn, VIC 3122, Australia.

## Abstract

Enhancing the hydrodynamic interfacial mobility of bubbles and droplets in multiphase systems is expected to reduce the characteristic coalescence times and thereby affect the stability of gas or liquid emulsions that are of wide industrial and biological importance. However, by comparing the controlled collision of bubbles or water droplets with mobile or immobile liquid interfaces, in a pure fluorocarbon liquid, we demonstrate that collisions involving mobile surfaces result in a significantly stronger series of rebounds before the rapid coalescence event. The stronger rebound is explained by the lower viscous dissipation during collisions involving mobile surfaces. We present direct numerical simulations to confirm that the observed rebound is enhanced with increased surface mobility. These observations require a reassessment of the role of surface mobility for controlling the dynamic stability of gas or liquid emulsion systems relevant to a wide range of processes, from microfluidics and pharmaceuticals to food and crude oil processing.

## Teaser

Mobile surface bubbles and droplets coalesce faster but can bounce back much more strongly when colliding.

## INTRODUCTION

The dynamic interaction and coalescence between deformable bubbles and droplets underpins the properties of colloidal systems that are central to a wide range of industrial applications and various naturally occurring and biological processes. Because of their practical relevance, the collision and coalescence between bubbles and droplets have been studied extensively by various experimental methods as well as by theoretical modeling and numerical simulations (*1*–*11*). Essentially, the outcome of the collision between two bubbles or droplets is determined by the short-ranged surface forces of chemical origin that act over the nanometer range and by the hydrodynamic interaction forces that act over the scale from bubble/droplet size down to contact. The handling and processing of emulsion systems are strongly dependent on the hydrodynamic interaction, which, in turn, strongly depends on the tangential mobility of the gas-liquid and liquid-liquid interfaces. Clean gas-liquid interfaces of bubbles are expected to obey the tangential stress-free or mobile hydrodynamic boundary condition in contrast to the no-slip or immobile boundary condition expected to hold at liquid-solid interfaces (section S1). Mobile surface bubbles and droplets are expected to coalescence much faster because of the lower hydrodynamic resistance during their approach. However, the experimental observation and quantification of the surface mobility effects have been problematic because even trace amounts of surface-active impurities, too small to be detected by interfacial tension measurements or in conductivity measurements in the case of water, can lead to complete immobilization of the bubble or droplet interface (*12*–*16*). Here, we conduct experiments in which a high-purity perfluorocarbon liquid is the continuous phase with viscosity close to that of water. We demonstrate a novel effect of enhanced bouncing due to increased surface mobility that can play a significant role in the practical control of colloidal system stability. Perfluorocarbon liquids have the distinct advantage that they have extremely low solubility for contaminants, thereby facilitating precise control of effects due to surface-active contaminants.

Because of the extreme sensitivity of surface mobility to contamination, particularly in the case of the most practically relevant liquid, water, there have been few detailed experimental studies to quantify the effects of surface mobility. One approach to demonstrate viscosity ratio effects on interface mobility and coalescence has been to use polymer droplets in immiscible polymer fluids, where both have viscosities three to five orders of magnitude higher than water (*17*, *18*). Recently, we have conducted a study (*19*) in which we use a perfluorocarbon liquid, PP11 [perfluoroperhydrophenanthrene (C_14_F_24_); F2 Chemicals], that has a viscosity about 20 times higher than water to evaluate the effects of the surface mobility. There, we study the free rise of gas bubbles with mobile surfaces or water droplets with immobile surfaces and their collision with a liquid interface. Here, we present new experiments using a lower-viscosity hydrocarbon liquid, PP1 [perfluoro-2-methylpentane (C_6_F_14_); F2 Chemicals]. It has a viscosity μ = 0.78 mPa·s, which is close to that of water and thus allows investigation of coalescence dynamics under practically relevant conditions. Furthermore, the interfacial tension of waterdrops in PP1 can be tuned to match that of bubbles in PP1 (σ = 12.4 mN/m) by the addition of surfactants so that surface tension effects on the collision and coalescence events can be isolated.

Our experiments focus on the collision between a rising bubble and a rising water droplet inside PP1, with an upper PP1-air or PP1-water interface. Both the PP1-air interface of the rising bubble and the upper PP1-air interface are shown to be tangentially mobile and stress-free, whereas the PP1-water droplet interface and the upper PP1-water interface turn out to be immobile or no-slip interfaces. To ensure that the water droplets and the upper PP1-water interface are immobile, we added the nonionic surfactant, Triton X-100, to the aqueous phase at a concentration of 2 × 10^−4^ M while also making the interfacial tension with PP1 take the same value of 12.4 mN/m as the PP1-air surface tension.

To quantify the difference in the collision dynamics at mobile or immobile fluid interfaces, we use a high-speed video camera at 5000 to 50,000 frames per second (fps) to record the collision and coalescence between a rising bubble and the various interfaces. The case of a bubble rising toward the free PP1-air interface corresponds to a mobile-mobile interface collision, and a bubble rising toward PP1-water interface corresponds to a mobile-immobile interface collision. Similarly, the case of water droplet rise toward free PP1-air interface corresponds to an immobile-mobile interface collision, and water droplet rising toward PP1-water interface corresponds to an immobile-immobile interface collision.

We measure the terminal velocities of rising bubbles and droplets due to buoyancy to confirm that the PP1-air bubble surface is mobile and the PP1-water droplet interface is immobile. The novel phenomenon of bubble and droplet bounce enhancement due to the mobility of the interface is done by observing collision events between the rising bubble and water droplets with a PP1-air and PP1-water interface. We also conduct direct numerical simulations (DNS) that replicate the surface mobility effects on the bubble and droplet bouncing dynamics. Last, we present simulation results that predict the outcomes of collision between two identical droplets with mobile or immobile interfaces.

## RESULTS

### Bubble and droplet terminal rise velocity

A simple way to evaluate the mobility of the PP1-air and PP1-water interfaces is to measure the dependence of the terminal velocity, *U*, of the free-rising bubbles and water droplets in PP1 on the Reynolds number, *Re* = ρ*DU*/μ, where ρ and μ are, respectively, the density and shear viscosity of the PP1 continuous phase and *D* is the bubble or droplet diameter (*19*, *20*). Mobile surface bubbles or droplets are expected to rise faster than those with immobile surfaces. For small Reynolds numbers (*Re* < 0.1), the mobile bubble rise velocity is expected to be 1.5 times higher than the immobile bubble rise velocity, given by the familiar Stokes’ law for solid spheres (*12*, *13*). However, for our experiments, the Reynolds numbers exceed the Stokes regime, and we cover two bubble size ranges: smaller bubbles that are spherical in shape (50 μm < *D* < 200 μm) and larger bubbles that take on an oblate ellipsoidal shape (200 μm < *D* < 1000 μm). In both cases, the bubble rise velocities closely followed the theoretical predictions for mobile interfaces from the study of Mei *et al.* (*21*) for the smaller undeformed spherical bubble size range (fig. S2) and the Moore theory (*22*) for deformed bubbles at higher *Re* > 50 (fig. S3). Measurements of the rise velocities of spherical water droplets (with or without added surfactant), 50 μm < *D* < 500 μm, follow the immobile-surface Schiller-Naumann empirical rule (*23*) observed for the motion of solid spheres. In summary, the free-rising bubbles and droplet terminal velocity experiments confirm that bubbles in PP1 have a fully mobile interface, whereas the surface of water droplets in PP1 is an immobile interface. Details of these measurements are given in section S3.

### Free-rising bubbles and droplets bouncing from mobile and immobile interfaces

We now compare the collision of free-rising bubbles traveling initially at their terminal velocity with the PP1-air interface or with the PP1-water with Triton X-100 solution interface of identical interfacial tensions of 12.4 mN/m. Smaller-size bubbles (*D* < 250 μm) reach the interface without bouncing back from it; however, larger-size bubbles bounce once (250 μm < *D* < 425 μm) or twice (*D* > 425 μm) before coalescence at the mobile PP1-air interface. On the other hand, at the PP1-water interface, the bubble rebounds once or twice, depending on its size, but eventually stops and remains intact without coalescence. The final outcome of the bubble-interface collision is determined by the sign of the van der Waals interaction between the bubble and the upper interface (*24*). The van der Waals force is attractive for the air-PP1-air system, and this results in the eventual coalescence of the bubble with the free surface. However, the van der Waals force is repulsive for the air-PP1-water system, and this results in the bubble coming to rest against the interface without coalescence.

**Fig. 1:**
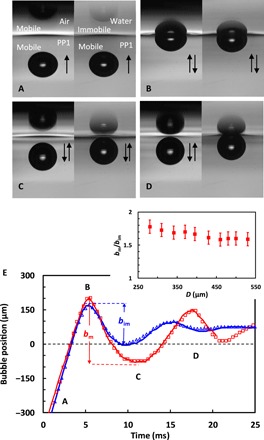
Bubble bounces from mobile and immobile interfaces. Movie S1 snapshots showing the bouncing of identical *D* = 480 μm undeformed-diameter bubbles from the mobile PP1-air interface (left) or from the immobile PP1–water solution interface (right): (**A**) approaching the interface, (**B**) first collision with the interface, (**C**) after the first bounce, and (**D**) after the second bounce. (**E**) Position of the bubble center of mass relative to the initial surface for the PP1-air (open red squares) or PP1–water solution (open blue triangles). The (A) to (D) snapshot positions are marked on the graph. The solid red line represents the DNS result for the bubble bounce from the PP1-air interface, shown in movie S3, and the blue solid line represents the DNS result for the bubble bounce from PP1–water solution interface, as shown in movie S4. The inset in (E) shows the dependence of the ratio between the bubble center-of-mass first bounce amplitude for the mobile interface and the first bounce amplitude for the immobile interface case, *b*_m_/*b*_im_, on the undeformed bubble diameter, *D*.

In all cases when the bubble bounces back from the interface, a significantly stronger rebound was observed at the mobile PP1-air interface compared to the immobile PP1-water interface. The difference in the bubble bounce kinetics can be seen in movie S1 and panels A to D of Fig. 1, which are snapshots from this movie. The case of a bubble, of 480 μm undeformed diameter, bouncing against the PP1-air interface (left side) or for an identical-sized bubble bouncing against the PP1-water interface (right side) is shown. The time dependence of the position of the center of mass of the bubble is extracted from the movies and given in Fig. 1E. The bubble bounces much stronger from the mobile PP1-air interface than from the immobile PP1-water interface. Numerous runs were conducted using bubbles in the range of 250 μm < *D* < 600 μm of undeformed bubble diameters, corresponding to Reynolds numbers of the rising bubbles of 40 < *Re* < 200. The reproducibility of the bubble bounce experiments was excellent, with <8-μm (two pixels in the video images) deviation in the measured first bounce amplitude between repeating runs using identical bubble diameters. The bounce enhancement effect was quantified as the ratio of the mobile to immobile interface first bounce amplitudes, *b*_m_/*b*_im_. The data for *b*_m_/*b*_im_ presented in Fig. 1E (inset) show a factor of 1.7 ± 0.1 increase in the bubble bounce amplitude, across the range of the studied bubble diameters.

We recognize that, together with interface mobility, there are other independent factors that might contribute to the bubble bouncing dynamics such as the variation in the upper-phase density and viscosity, e.g., air versus water phase. The upper-phase density-related gravity effect in comparison to the capillary force contribution for the bubble bounce from the interface can be estimated from the value of the Bond number, *Bo* = ∆ρ*gR*^2^/σ, where Δρ is the density difference between the lower and upper phases, *R* is the bubble radius, σ is the interfacial tension, and *g* is the gravity constant. For bubble bounce from the PP1-air interface, *Bo* < 0.12 for 2*R* < 600 μm, and for the bounce from the PP1-water interface, *Bo* < 0.05 for 2*R* < 600 μm. The small values of the Bond numbers indicate that density difference effects are unlikely to significantly affect the way the bubble bounces from the interface.

**Fig. 2:**
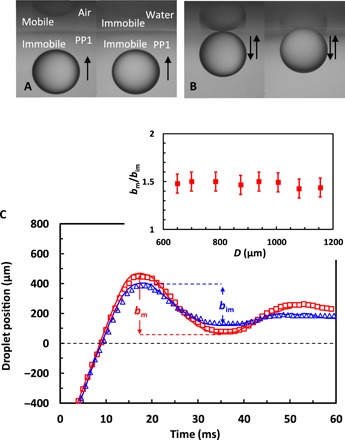
Droplet bounces from mobile and immobile interfaces. Movie S2 snapshots showing the bouncing of a 1080-μm water solution droplet from the PP1-air interface (left) or the PP1–water solution interface (right): (**A**) approaching the interface and (**B**) after the first bounce. (**C**) Position of the droplet center of mass relative to the undisturbed interface for the PP1-air (open red squares) or PP1–water solution (open blue triangles) interface bounces. The solid blue line represents the DNS result for the droplet bounce from the PP1–water solution interface shown in movie S5, and the solid red line represents the DNS result for the droplet bounce from the free PP1-air interface shown in movie S6. The inset shows the dependence of the ratio between the droplet center-of-mass first bounce amplitude for the mobile interface and the first bounce amplitude for the immobile interface case, *b*_m_/*b*_im_, on the undeformed droplet diameter, *D*.

Furthermore, the same trend for a stronger bounce from the mobile PP1-air interface, compared to immobile PP1-water interface of identical interfacial tension, was observed when water solution droplets collided with these interfaces. This is demonstrated in movie S2, which compares the bounce of a 1080-μm water solution droplet from the PP1-air with that from a PP1–water solution interface. Figure 2 (A and B) shows snapshots from this movie, and Fig. 2C shows the motion of the droplet center of mass. The enhanced bouncing effect is somewhat less strongly pronounced compared to the bubble bouncing case, which can be due to lower rise velocities of the droplets compared to the bubbles in these experiments. Nevertheless, systematic differences in the bounce were observed using various droplet sizes in the range between 600 μm < *D* < 1200 μm, corresponding to Reynolds number of the rising droplets 60 < *Re* < 270. The inset in Fig. 2C quantifies this effect in terms of the enhancement of the ratio of the mobile to immobile interface first bounce amplitude, *b*_m_/*b*_im_, with a value of 1.5 ± 0.1 across the entire range of the droplet sizes investigated.

We note that for the droplet experiments, the Bond number is larger compared to the bubble experiments because of the larger droplet diameters used. For the example given in Fig. 2, *2R* = 1080 μm, *Bo* = 0.34 for the PP1-air interface, and *Bo* = 0.14 for the PP1-water. As the Bond number is less than 1, the effect of gravity is not expected to make a very significant contribution to the decrease of the bounce amplitude from the PP1-water interface.

In summary, the bubble and droplet bounce experiments indicate that collisions involving mobile interfaces can produce stronger rebounds than those involving immobile interfaces. The collision between mobile-mobile interfaces produces the strongest rebound, and that between immobile-immobile interfaces produces the weakest rebound. The physics of the bubble bounce phenomenon can be considered in terms of a part of the kinetic energy of the fluid around the rising bubble being converted into surface and potential energy associated with surface deformations during the collision, which is subsequently transformed back into the kinetic energy of the rebounding bubble (*25*). Consecutive bounces are weaker because of viscous dissipation in the liquid between the bubble and interface during the collision. As there is less stress and less related viscous dissipation for mobile surfaces, the bubble produces more surface deformation and bounces stronger from a mobile interface. To further evaluate this effect, we conducted a series of numerical simulations.

### Coalescence time scale for mobile and immobile interfaces

Although the above qualitative explanation of stronger bounces from mobile surfaces is sound, predicting such a phenomenon may perhaps be counterintuitive to the expectation that two mobile-surface droplets or bubbles would coalesce much faster compared to immobile surfaces because of the much lower hydrodynamic resistance in the thin liquid film separating bubbles before the final coalescence occurs. Two mobile surfaces of droplets or bubbles, which coalesce without bouncing back, will produce a much faster coalescence, as shown in prior experiments using higher-viscosity liquids (*17*–*19*).

**Fig. 3:**
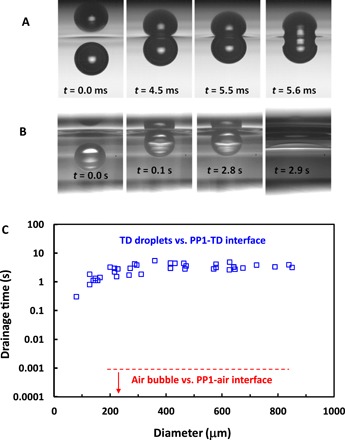
Characteristic drainage times. Chronographic snapshots of (**A**) 410-μm bubble coalescence with the PP1-air interface following an initial rebound from the interface and (**B**) a 660-μm tetradecane (TD) oil droplet coalescence with the PP1-tetradcane interface. Time is listed in each snapshot. (**C**) Dependence of the drainage time on the droplet diameter for tetradecane oil droplets versus PP1-tetradecane interface (open blue squares). For reference, the bubble versus PP1-air drainage time of less than 1 ms is indicated (below the dashed red line).

The faster coalescence for mobile surfaces was also confirmed by the present experiments, if there is no rebound. In this case, for the low-viscosity PP1, the bubble seems to coalesce almost instantaneously with the interface. The time scale of the coalescence, which is roughly the time that the bubble or droplet spends next to the interface before coalescing, is determined by the drainage time of the thin liquid film that separates the bubble or droplet from the interface. The drainage times for bubbles coalescing with free PP1 surface were found to be several orders of magnitude shorter than for a water or tetradecane oil droplet coalescing with PP1-liquid interfaces, which are examples of immobile-surface coalescence. This orders of magnitude difference in drainage times is illustrated in Fig. 3, for the case of tetradecane droplets coalescing with PP1-tetradecane interface. The snapshots in Fig. 3A, taken using a high filming rate of 40,000 fps, show the almost-instantaneous coalescence of the bubble with the PP1-air interface (less than 1 ms), representative of the coalescence of two mobile surfaces. In comparison, the snapshots of a tetradecane oil droplet coalescing with PP1-tetradecane interface (Fig. 3B) and the drainage time results (Fig. 3C) are representative of immobile-surface coalescence that shows three orders of magnitude longer drainage times from submilliseconds to seconds. Further details on the drainage time determination are given in section S4.

### Gerris numerical simulations

DNS of the process of deformable bubble and droplets approach, bounce, and coalescence is a complex hydrodynamic problem because the different processes involved span changes over several orders of magnitudes in the separation distance and time scales. The simplicity and well-controlled boundary conditions of our experiments provide an excellent opportunity to test the present capabilities of the DNS using the freely available code Gerris (*26*–*32*). This code uses the volume-of-fluid (VOF) method to solve the incompressible Navier-Stokes equations. To simulate the bubble or droplet bounce problem, the solver uses axisymmetric geometry, which optimizes the computational cost. Its high degree of parallelization and dynamic adaptive grid refinement allow us to resolve the entire process of bubble or droplet release from below the surface, its acceleration to reach terminal velocity, and subsequent bouncing from the interface.

For the simulation of the bubble rising toward the PP1-air interface and water droplet rising to water-PP1 interface, we use the generic Gerris two-phase VOF method. To simulate the bubble bounce from the PP1-water interface and droplet bounce from the PP1-air interface, we use a three-phase VOF method, following the approach given by Chen *et al.* (*30*). This approach allows us to use the existing capabilities of the Gerris code to include a top phase with density and viscosity that can be different from both the continuous phase and that of the rising bubble or the droplet.

A direct comparison between simulation and experimental observation of a bubble collision and bounce from the free PP1 interface is given in movie S3. The spatial and temporal parameters of the simulation are identical to those of the experimental system. The agreement between the two is excellent in every detail. The red solid line in Fig. 1E quantified the agreement for the bubble position versus time, supporting the visual results in movie S3. We note that to accurately capture the minute liquid film, 16 levels of localized grid refinement are required. To the authors’ knowledge, there is no proven theoretical modeling approach that can quantitatively predict the mobile bubble bounce from a deformable mobile interface.

Gerris does not allow the direct application of the no-slip boundary condition on the surface of the droplet or PP1-water interface, as the default surface mobility is determined by the two-phase viscosity ratio. However, by using a 10-fold higher water phase viscosity, we were able to effectively simulate a near–no-slip boundary condition at the PP1-water interfaces. The solid blue line in Fig. 1E shows the result for the three-phase simulation of the bubble bouncing from the PP1-water interface visualized in movie S4. A very good agreement with the experiment is found when water density and a 10-fold water viscosity were assigned for the top phase (see also fig. S5 including simulation results for both the original water viscosity and 10-fold water viscosity).

The same approach of using a 10-fold higher water viscosity for the water phase was used both in the two-phase simulation of the water droplet bouncing from the PP1-water interface shown in movie S5 and in the three-phase simulation of water droplets bouncing from the PP1-air interface shown in movie S6. The solid blue and red lines in Fig. 2C give respective center-of-mass simulation results showing very reasonable agreement with the experiments.

**Fig. 4:**
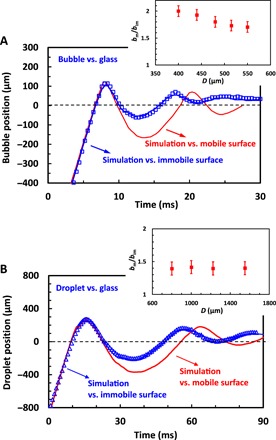
Bubble and droplet bounces from solid interfaces. (**A**) Comparison of experimental data for center-of-mass position versus time for the collision of a 480-μm bubble, rising in PP1, with a flat glass surface (open blue squares; movie S7, left) with DNS results for the bubble bouncing from the no-slip solid flat (solid blue line; movie S7, middle) or a free-slip solid flat (solid red line; movie S7, right). The inset shows the DNS results for the enhanced bouncing amplitude, *b*_m_/*b*_im_, as a function of the bubble diameter. (**B**) Comparison of experimental data for center-of-mass position versus time for the collision of a 1550-μm water-glycerol mixture droplet rising in PP1 with a flat glass surface (open blue triangles; movie S8, left) with DNS results for the droplet bounce from the no-slip solid flat (solid blue line; movie S8, middle) or a free-slip solid flat (solid red line; movie S9, right). The inset shows the DNS results for the enhanced bouncing amplitude, *b*_m_/*b*_im_, as a function of the droplet diameter.

The good agreement of simulations with experiments, in all studied cases, gives us confidence to further evaluate the effect of surface mobility by conducting additional numerical simulations. As discussed above, other factors that can influence the strength of the bubble or droplet bouncing from the interface are the variation of density and viscosity in the upper phase. An alternative way to evaluate the surface mobility effect in the bubble or droplet bouncing experiments, which is completely decoupled from the upper phase properties, is to compare simulation for the bouncing from a flat solid free-slip and a flat solid no-slip interface that can be readily done with Gerris. Movie S7 compares the experimental observations of a 480-μm bubble bouncing from a flat glass interface (left) with simulation of the bubble bounce from a no-slip solid flat interface (middle) or free-slip flat interface (right). In Fig. 4A, we compare the corresponding bubble center-of-mass position versus time. There is excellent agreement between experiment and simulation of the bounce from the immobile interface. The simulation for the bubble bounce from the free-slip mobile interface shows the same magnitude of the bubble bounce enhancement as in our deformable interface experiments. Simulations using several different bubble sizes confirm the trend for a stronger bounce from the mobile interface, with a *b*_m_/*b*_im_ factor between 1.7 and 2.0 as presented in the inset of Fig. 4A.

In the case of a water droplet bouncing from a glass surface, we use a 46 volume percent (volume %) water and a 54 volume % glycerol mixture for the droplet of viscosity, which matches the simulation, i.e., 10-fold water viscosity. We also use the droplet without added surfactant having an interfacial tension against PP1 of 43.4 mN/m to enhance the droplet bounce from the interface. As in the case of the bubble bounce, the simulations show an excellent agreement between immobile interface bounce and experiments, as can be seen in movie S8 and Fig. 4B, which compares the droplet center-of-mass positions versus time. Simulations for the bounce from a free-slip flat interface show a bounce enhancement, *b*_m_/*b*_im_ = 1.4 ± 0.1 for the range of different droplet sizes presented in the inset of Fig. 4B. This ratio is only slightly lower than for the case of droplets bouncing from a deformable interface.

The simulations of bubble and droplet bounces from a solid interface confirm the mobile-interface bouncing enhancement effect that is independent of the upper-phase properties. The close magnitude of the effect shown in these simulations to that in the deformable-interface experiments, together with the Bond number estimate, makes a strong case that the interface mobility is the leading effect for the bouncing enhancement demonstrated in our experiments.

### Simulations of the collision of two droplets

So far, all experimental observations and simulation studies pertain to the collisions of bubbles or droplets that are rising under a constant buoyancy force. To further investigate the implication of the surface mobility effects demonstrated in the above studies for bouncing from deformable interfaces, we performed simulation of the collision of identical droplets of low and high interfacial mobility. Because of the obvious symmetry, the collision of two equal-sized droplets is identical to that of a droplet with a free-slip solid surface, which can be readily simulated using Gerris to predict the outcome of these collision events.

Initial acceleration of the droplets was performed identically as in the simulations of freely rising buoyancy-driven droplets. However, once the droplet reached within 1 or 2 radii (1*R* or 2*R*, *R* = *D*/2) from the surface, gravity was switched off. The subsequent simulation is therefore representative of the free collision between two identical droplets in the absence of any external forces. To make the bounce effect more pronounced, we used 1.2-mm-diameter droplets with an interfacial tension equal to that between PP1and pure water, σ = 55.0 mN/m. Using droplets with viscosity equal to that of water, we simulate a collision of higher–surface mobility droplets, and using droplets with 10 times the water viscosity, we simulated droplet with low interfacial mobility.

Movies S9 and S10 give examples of the results for the collisions between high– or low–surface mobility droplets. In the case of movie S9, gravity was removed when the surface of the droplet is at 2*R* from the symmetry surface (this corresponds to a droplet separation of 4*R*), and for movie S10, gravity was removed when the surface of the droplet is at 1*R* (corresponding to a separation of 2*R* between droplets). As can be expected from the earlier experiments for bouncing from an initially flat but deformable interface, simulations in movies S9 and S10 demonstrate that in the initial encounter before coalescence, the higher-mobility droplets bounce further apart compared to the low-mobility droplets. This is due to both higher initial velocity acquired when identical acceleration fields are applied and lower dissipation losses during the collision.

**Fig. 5:**
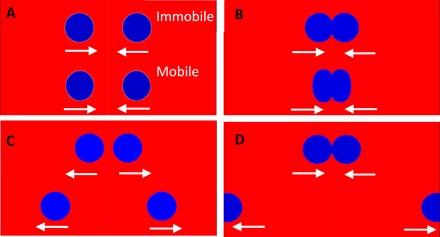
Simulations of colliding droplets. Snapshots from movie S10 comparing the simulation of the upper pair of low-mobility interface water droplets (×10 water viscosity) and the lower pair of the high-mobility interface water droplets (×1 water viscosity) colliding inside PP1. Droplet diameter is 1.2 mm, and water-PP1 interfacial tension is 55 mN/m. Droplets are initially accelerated by gravity to reach terminal velocity. Gravity is removed at 2*R* droplet separation to simulate the external force–free collision. The arrows show the direction of droplet momentum: (**A**) at the moment of gravity removal, (**B**) collision of the droplets, and (**C**) droplet bounce back after colliding. (**D**) The low-mobility droplets move back to collide a second time and eventually coalesce, while the high-mobility droplets continue to move further apart without a reencounter.

Apart from the stronger bounce, in the case of mobile-surface droplets, another intriguing phenomenon observed in these simulations is demonstrated in movie S10 for the low–surface mobility collision. In this case, after the first collision, the droplets initially separate but then move back to collide a second time even in the absence of any external forces driving the droplets together. This is schematized in the snapshots from the movie shown in Fig. 5. Using the same refinement level, reflecting a critical film thickness at which the droplets are allowed to coalesce, the final outcome from the collision shown in movie S10 is a coalescence of the immobile droplets during the secondary collision, whereas the mobile droplets bounce apart and continue to separate without coalescing.

**Fig. 6:**
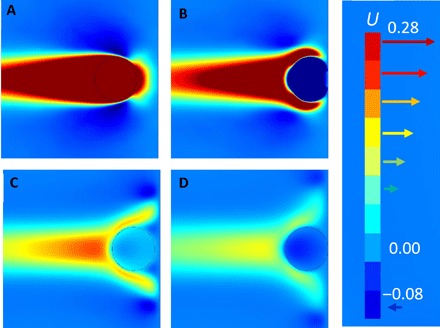
Velocity field of colliding immobile-surface droplets. Snapshots from movie S11 visualizing the horizontal component of the velocity field in the case of *D* = 1.2 mm and ×10 water viscosity droplet collision with gravity removed at 2*R* droplet separation. We show only one of the droplets, which collides at the right edge of the frames: (**A**) at the moment when gravity is removed, (**B**) during the first collision, (**C**) instant of reversal for second collision, and (**D**) just before coalescence during the second collision. The color scale on the right shows the dimensionless magnitude of the horizontal velocity.

A qualitative explanation of the droplet secondary collision phenomenon is given by movie S11, which provides a visualization of the velocity field during the collision, with snapshots shown in Fig. 6. As seen in Fig. 6A, during the droplet acceleration by the gravity field, a well-pronounced wake of liquid trailing the droplet is formed. Following the first collision (Fig. 6B), the inertia of the liquid in the wake opposes the droplet rebound and eventually reverses its motion (Fig. 6C) to promote a second collision (Fig. 6D). In essence, this is an added mass effect as one takes into account the momentum of the surrounding liquid. As seen by comparing movies S9 and S10, this effect results in a second collision only at certain parameter values and is sensitive to factors such as the droplet acceleration history. The exact condition under which these rebound and reencounter, in the absence of an external driving force, requires future quantitative studies supported by experiments. However, it is clear that the simple idea of treating colliding drops and bubbles as effective particles without cognizance of the behavior of the surrounding continuous phase can overlook such subtle behavior of a complex system.

## DISCUSSION

Interfacial mobility is a well-known factor that predetermines the hydrodynamic interaction between deformable bubbles and droplets. Traditionally, higher surface mobility has been anticipated to lead to much faster coalescence due to the lower hydrodynamic resistance for the drainage of the thin liquid film separating the bubbles or droplets before coalescence. However, assessing the exact role of the surface mobility on the entire process of bubble and droplet collisions has been problematic, both because of the experimental challenges in controlling the interface mobility and because of the complexity of the general coalescence theory. In practical systems, the situation is further complicated by the presence of interfacial forces such as van der Waals and electric double layer and various specific interactions, which makes it difficult to quantify the role of surface mobility.

To determine the specific effect of the surface mobility on the collision dynamics, we have conducted experiments with bubbles in pure perfluorocarbon liquid, PP1, as model of a mobile-interface deformable particulate, and water droplet in PP1, as model of immobile-interface deformable particulates. Furthermore, we can adjust the PP1-water interfacial tension to match the PP1-air surface tension creating mobile and immobile interfaces with identical deformability.

In all cases when the bubble bounces from the interface without coalescing, a much stronger bounce was observed from a mobile interface (about two times larger in amplitude) compared to an immobile interface with identical deformability. The same trend was observed for the bouncing of water droplets from the interfaces. These observations were reproduced in our numerical simulations with high fidelity, thus giving us confidence to use the numerical model to understand the role of the continuous phase in causing the rebound and reencounter phenomenon between colliding bubbles or droplets that are not under external forces.

The good agreement between experiments and numerical simulations can be further applied to advance theoretical modeling of mobile and immobile bubbles and droplet collisions. While the numerical simulations can be very accurate, they are computationally intensive because of the thin film, which requires extreme grid refinement, and the simulations do not provide direct insight into the underlying physical mechanisms. On the other hand, theoretical modeling of bubble and droplet coalescence has been developing rapidly during the past decades (*6*–*10*). This approach can potentially give direct insight into the underlying physical model and system parameters that control the interaction in the thin film. The challenge is to include the effects of the continuous phase beyond perhaps the use of a simple effective mass contribution and to account for the effects of mobile or immobile hydrodynamic boundary conditions at deformable interfaces (*6*). Present experiment with well-defined boundary condition and matching numerical simulations is a perfect model system to further advance the theoretical treatment.

As demonstrated in our droplet pair bounce simulations, DNS can provide detailed information for the transfer of momentum between the phases that can be difficult to account for using theoretical modeling. Simulation results can be further used to determine the transfer of energy between the phases and relate it to the variations in the interface mobility. One area in which the present simulation can be further improved is to enable the application of the no-slip boundary condition on the fluid-fluid interface, which will reflect the interface immobilization by the surface-active agents.

The enhanced bouncing effect has diverse implications for the dynamic stability of colloidal systems involving gas-liquid and liquid-liquid interfaces. From our study, it follows that the accumulative effect from the changing droplet or bubble surface mobility is a competition between enhanced bounce kinetics and decrease in the drainage times. For example, in a system where the coalescence rate is low once the system is mechanically agitated, settling will occur faster for immobile interface droplets or bubbles, as illustrated in our droplet collision simulations. On the other hand, for slower, more static systems, the predominant effect will be the faster drainage times for mobile interfaces.

In practical systems, the surface mobility can be regulated by the addition of surface-active substances. Very small bubble or oil droplets are expected to be immobilized even by trace contaminations; however, for larger droplets and bubbles and in system with higher applied shear rates, the surface mobility effects are readily observed (*32*–*34*), and the present investigation can direct novel approaches to manipulate the dynamic stability and performance of these systems.

## MATERIALS AND METHODS

### Perfluorocarbon liquid and water surfactant solution

The perfluorocarbon liquid used was Flutec PP1, High-Performance Fluid, from F2 Chemicals Ltd., which is mostly composed of C_6_F_14_. The PP1 density is ρ = 1.71 g/cm^3^, and dynamic viscosity was measured to be μ = 0.78 mPa·s. The water phase was a water solution of Triton X-100 surfactant at a concentration of 2 × 10^−4^ M. The surface tension of the PP1-air liquid interface was measured to be 12.4 mN/m, which can be matched with PP1-water solution of 2 × 10^−4^ M Triton X-100 with an interfacial tension of about 12.4 mN/m.

### Experimental design and protocol

A schematic of the experimental setup is shown in fig. S1. Bubbles or water droplets were released from a fine-end microcapillary mounted close to the bottom of a glass container (2.5 × 2.5 cm; height, 7.5 cm) that was two-thirds filled with PP1. The phase above the PP1 was either air or the water solution of 2 × 10^−4^ M Triton X-100 to furnish a PP1-air or PP1–water solution interface above the rising bubble or water droplet of equal interfacial tension. Bubble and water droplets of diameters *D* = 50 to 1000 μm were produced using different-sized microcapillaries. The free rise and collision of a bubble or water droplet with the upper PP1-air or PP1-water surface were monitored using a high-speed camera (Photron SA5) equipped with a microscope with 5× or 10× objective and using a frame rate of between 5000 and 50,000 fps.

To confirm the PP1-air interface mobility, we measured the terminal rise velocity of air bubbles and, for PP1-water interface, the terminal rise velocity of water solution droplets. To observe the effect of the surface mobility on the bounce dynamics, we compared the bounce of free-rising bubbles from the PP1-air interface corresponding to the mobile-mobile interface collision case with identical-sized free-rising bubbles for the PP1–water solution interfaces corresponding to mobile-immobile interface collision case. Respectively, using free-rising water solution droplets, we compared the bounce from PP1-air corresponding to the immobile-mobile interface case to the bounce from PP1–water solution case corresponding to immobile-immobile interface collision case. Complete experimental and numerical simulation details can be found in the Supplementary Materials.

## Supplementary Material

http://advances.sciencemag.org/cgi/content/full/5/10/eaaw4292/DC1

Download PDF

Movie S1

Movie S2

Movie S3

Movie S4

Movie S5

Movie S6

Movie S7

Movie S8

Movie S9

Movie S10

Movie S11

Mobile-surface bubbles and droplets coalesce faster but bounce stronger

## SUPPLEMENTARY MATERIALS

Supplementary material for this article is available at http://advances.sciencemag.org/cgi/content/full/5/10/eaaw4292/DC1 Section S1. Mobile and immobile liquid interfaces Section S2. Experimental details Section S3. Bubble and droplet terminal rise velocity Section S4. Drainage time experiments details Section S5. Gerris DNS Fig. S1. Experimental setup and schematics. Fig. S2. Rise velocity for spherical bubbles and droplets. Fig. S3. Rise velocity for larger bubbles. Fig. S4. Computational domain and adaptive mesh. Fig. S5. Comparison between experiment and simulation. Movie S1. This combined movie shows the bouncing of a bubble of 480 μm undeformed diameter from the free PP1-air interface (left) or the PP1–water solution interface (right) of equal deformability. Movie S2. This combined movie shows the bouncing of a 1080-μm water solution droplet from the PP1-air interface (left side) or the PP1–water solution interface (right side) of equal deformability. Movie S3. This movie compares experiment (left) with simulation result (right) for the bouncing of a 480–μm–undeformed-diameter bubble from the free PP1-air interface. Movie S4. This movie compares experiment (left) with simulation result (right) for the bouncing of a 480–μm–undeformed-diameter bubble from the PP1–water solution interface. Movie S5. This movie compares experiment (left) with simulation (right) for the bouncing of a 1080–μm–undeformed-diameter water solution droplet from the PP1–water solution interface. Movie S6. This movie compares experiment (left) with simulation (right) for the bouncing of a 1080–μm–undeformed-diameter water solution droplet from the free PP1-air interface. Movie S7. This movie compares experiment (left) for the bouncing of a bubble of 480 μm undeformed diameter free-rising in PP1 from a flat glass surface with simulation for the bubble bounce from no-slip solid flat (middle) or a free-slip solid flat (right). Movie S8. This movie compares experiment (left) for the bouncing of a water-glycerol mixture droplet of 1550 μm undeformed diameter free-rising in PP1 from a flat glass surface with simulation for the droplet bounce from no-slip solid flat (middle) or a free-slip solid flat (right). Movie S9. Simulation for the collision of the two pure water droplets of 1.2 mm undeformed diameter in PP1 for the case of low-mobility water droplets (10× water viscosity, the upper pair) or high-mobility water droplets (1× water viscosity, the lower pair). Movie S10. Simulation for the collision of the two pure water droplets of 1.2 mm undeformed diameter in PP1 for the case of low-mobility water droplets (10× water viscosity, the upper pair) or high-mobility water droplets (1× water viscosity, the lower pair). Movie S11. Velocity field visualization in the simulation of the collision of the two 1.2-mm pure water droplets in PP1 of low surface mobility with gravity switched off at 2*R* droplet separation (movie S10, top droplet pair case).
