# Supplementary material for: Mobile-surface bubbles and droplets coalesce faster but bounce stronger
Source: Sci Adv. 2019 Oct 25;5(10):eaaw4292. doi: 10.1126/sciadv.aaw4292 (PMC6814372; doi:10.1126/sciadv.aaw4292)
Supplement: http://advances.sciencemag.org/cgi/content/full/5/10/eaaw4292/DC1 [file supp_5_10_eaaw4292__index.html]

Science Advances | Science AdvancesAAASSearchScience AdvancesMenu

## Supplementary Materials

**The PDF file includes:**

- Section S1. Mobile and immobile liquid interfaces
- Section S2. Experimental details
- Section S3. Bubble and droplet terminal rise velocity
- Section S4. Drainage time experiments details
- Section S5. Gerris DNS
- Fig. S1. Experimental setup and schematics.
- Fig. S2. Rise velocity for spherical bubbles and droplets.
- Fig. S3. Rise velocity for larger bubbles.
- Fig. S4. Computational domain and adaptive mesh.
- Fig. S5. Comparison between experiment and simulation.
- Legends for movies S1 to S11

Download PDF

**Other Supplementary Material for this manuscript includes the following:**

- Movie S1 (.mov format). This combined movie shows the bouncing of a bubble of 480 μm undeformed diameter from the free PP1-air interface (left) or the PP1–water solution interface (right) of equal deformability.
- Movie S2 (.mov format). This combined movie shows the bouncing of a 1080-μm water solution droplet from the PP1-air interface (left side) or the PP1–water solution interface (right side) of equal deformability.
- Movie S3 (.mov format). This movie compares experiment (left) with simulation result (right) for the bouncing of a 480–μm–undeformed-diameter bubble from the free PP1-air interface.
- Movie S4 (.mov format). This movie compares experiment (left) with simulation result (right) for the bouncing of a 480–μm–undeformed-diameter bubble from the PP1–water solution interface.
- Movie S5 (.mov format). This movie compares experiment (left) with simulation (right) for the bouncing of a 1080–μm–undeformed-diameter water solution droplet from the PP1–water solution interface.
- Movie S6 (.mov format). This movie compares experiment (left) with simulation (right) for the bouncing of a 1080–μm–undeformed-diameter water solution droplet from the free PP1-air interface.
- Movie S7 (.mov format). This movie compares experiment (left) for the bouncing of a bubble of 480 μm undeformed diameter free-rising in PP1 from a flat glass surface with simulation for the bubble bounce from no-slip solid flat (middle) or a free-slip solid flat (right).
- Movie S8 (.mov format). This movie compares experiment (left) for the bouncing of a water-glycerol mixture droplet of 1550 μm undeformed diameter free-rising in PP1 from a flat glass surface with simulation for the droplet bounce from no-slip solid flat (middle) or a free-slip solid flat (right).
- Movie S9 (.mov format). Simulation for the collision of the two pure water droplets of 1.2 mm undeformed diameter in PP1 for the case of low-mobility water droplets (10× water viscosity, the upper pair) or high-mobility water droplets (1× water viscosity, the lower pair).
- Movie S10 (.mov format). Simulation for the collision of the two pure water droplets of 1.2 mm undeformed diameter in PP1 for the case of low-mobility water droplets (10× water viscosity, the upper pair) or high-mobility water droplets (1× water viscosity, the lower pair).
- Movie S11 (.mov format). Velocity field visualization in the simulation of the collision of the two 1.2-mm pure water droplets in PP1 of low surface mobility with gravity switched off at 2*R* droplet separation (movie S10, top droplet pair case).

**Files in this Data Supplement:**

- Adobe PDF - aaw4292\_SM.pdf
