# Supplementary material for: Mobile-surface bubbles and droplets coalesce faster but bounce stronger
Source: Sci Adv. 2019 Oct 25;5(10):eaaw4292. doi: 10.1126/sciadv.aaw4292 (PMC6814372; doi:10.1126/sciadv.aaw4292)
Supplement: Download PDF [file aaw4292_SM.pdf]

Science Advances
AAAS

advances.sciencemag.org/cgi/content/full/5/10/eaaw4292/DC1

Supplementary Materials for

Mobile-surface bubbles and droplets coalesce faster but bounce stronger

Ivan U. Vakarelski*, Fan Yang, Yuan Si Tian, Er Qiang Li, Derek Y. C. Chan, Sigurdur T. Thoroddsen

*Corresponding author. Email: ivanuriev.vakarelski@kaust.edu.sa

Published 25 October 2019, *Sci. Adv.* **5**, eaaw4292 (2018)
DOI: 10.1126/sciadv.aaw4292

The PDF file includes:

Section S1. Mobile and immobile liquid interfaces
Section S2. Experimental details
Section S3. Bubble and droplet terminal rise velocity
Section S4. Drainage time experiments details
Section S5. Gerris DNS
Fig. S1. Experimental setup and schematics.
Fig. S2. Rise velocity for spherical bubbles and droplets.
Fig. S3. Rise velocity for larger bubbles.
Fig. S4. Computational domain and adaptive mesh.
Fig. S5. Comparison between experiment and simulation.
Legends for movies S1 to S11

Other Supplementary Material for this manuscript includes the following:

(available at advances.sciencemag.org/cgi/content/full/5/10/eaaw4292/DC1)

Movie S1 (.mov format). This combined movie shows the bouncing of a bubble of 480 μm undeformed diameter from the free PP1-air interface (left) or the PP1–water solution interface (right) of equal deformability.
Movie S2 (.mov format). This combined movie shows the bouncing of a 1080-μm water solution droplet from the PP1-air interface (left side) or the PP1–water solution interface (right side) of equal deformability.
Movie S3 (.mov format). This movie compares experiment (left) with simulation result (right) for the bouncing of a 480–μm–undeformed-diameter bubble from the free PP1-air interface.
Movie S4 (.mov format). This movie compares experiment (left) with simulation result (right) for the bouncing of a 480–μm–undeformed-diameter bubble from the PP1–water solution interface.

Movie S5 (.mov format). This movie compares experiment (left) with simulation (right) for the bouncing of a 1080–μm–undeformed-diameter water solution droplet from the PP1–water solution interface.

Movie S6 (.mov format). This movie compares experiment (left) with simulation (right) for the bouncing of a 1080–μm–undeformed-diameter water solution droplet from the free PP1-air interface.

Movie S7 (.mov format). This movie compares experiment (left) for the bouncing of a bubble of 480 μm undeformed diameter free-rising in PP1 from a flat glass surface with simulation for the bubble bounce from no-slip solid flat (middle) or a free-slip solid flat (right).

Movie S8 (.mov format). This movie compares experiment (left) for the bouncing of a water-glycerol mixture droplet of 1550 μm undeformed diameter free-rising in PP1 from a flat glass surface with simulation for the droplet bounce from no-slip solid flat (middle) or a free-slip solid flat (right).

Movie S9 (.mov format). Simulation for the collision of the two pure water droplets of 1.2 mm undeformed diameter in PP1 for the case of low-mobility water droplets (10× water viscosity, the upper pair) or high-mobility water droplets (1× water viscosity, the lower pair).

Movie S10 (.mov format). Simulation for the collision of the two pure water droplets of 1.2 mm undeformed diameter in PP1 for the case of low-mobility water droplets (10× water viscosity, the upper pair) or high-mobility water droplets (1× water viscosity, the lower pair).

Movie S11 (.mov format). Velocity field visualization in the simulation of the collision of the two 1.2-mm pure water droplets in PP1 of low surface mobility with gravity switched off at $2R$ droplet separation (movie S10, top droplet pair case).

Section S1. Mobile and immobile liquid interfaces

Fluid molecules adjacent to a solid surface are expected to move with the same velocity as the surface in both the normal and tangential directions. This is often referred to as the no-slip or stick boundary condition. We will refer to this as an *immobile* interface or an *immobile* hydrodynamic boundary condition. On the other side, it is assumed that a clean gas-liquid interface cannot sustain any shear stress and this is referred as a fully *mobile* interface. For a clean liquid-liquid interface the tangential stress is continuous and the related mobility is determined by the ratio of the two liquid viscosities. A high viscosity droplet in low viscosity liquid will have surface mobility close to that on a solid surface, and a low viscosity droplet in high viscosity fluid will have a nearly mobile interface.

The immobile interface is also referred as no-slip boundary condition and tangentially mobile interfaces with zero shear stress as free-slip boundary condition. Moreover, the complex hydrodynamic condition at a solid surface that possesses small scale geometric structures or has been treated with a thin coating of adsorbates or has adsorbed gas bubbles, it is often subsumed in the notion of a *partially mobile* surface characterized by a phenomenological *slip length*.

In practice, the bubble or droplet interface could easily be contaminated by surface active contamination leading to immobilization of the interface. A more detailed review on the experiments showing the effect of the surface mobility on bubbles and droplet coalescence dynamics could be found in reference (*19*).

Section S2. Experimental details

The liquid used was the perfluorocarbon liquid FLUTEC© PP1, High Performance fluid from F2 Chemicals Ltd., that is mostly composed of perfluoro-2-methylpentane (C_6F_{14}). The PP1 liquid is clear and colorless with density, ρ = 1.71 g/cm^3. The PP1 dynamic viscosity as measured with an Ubbelohde capillary viscometer was found to be, μ = 0.78 mPa s, slightly lower than the value of μ = 1.10 mPa s given by the manufacturer. All experiments were conducted at the laboratory temperature of about 23 °C.

The aqueous phase in our experiments used to generate the water droplets in PP1 or as a top phase over the PP1 in the container to create a flat immobile interface was a water solution of the non-ionic surfactant Triton X-100 (Sigma-Aldrich) at concentration of 2×10^{-4} M. This concentration was selected to match the PP1-water solution interfacial tension of 12.4 ± 0.1 mN/m to the PP1-air interfacial tension of 12.4 ± 0.1 mN/m and is slightly below the critical micelle concentration of Triton X-100 of about 3×10^{-4} M. Various surface and interfacial tensions were measured with a Krüss tensiometer: PP1-air of 12.4 ± 0.1 mN/m, PP1-water (without surfactant) of 55.3 ± 0.1 mN/m; PP1-water with 1×10^{-4} M Triton X-100 of 18.0 ± 0.1 mN/m, PP1-water with 2×10^{-4} M Triton X-100 of 12.4 ± 0.1 mN/m; and PP1-water with 3×10^{-4} M Triton X-100 of 10.6 ± 0.1 mN/m. We also measured the PP1-tertadecane interfacial tension of 3.6 ± 0.1 mN/m. Tetradecane (Aldrich, 99.0+% olefine free) in our experiments was used as received. The 46 vol. % water and 54 vol. % glycerol mixture droplet, used in the experiments for droplets bouncing from the glass surface, has dynamic viscosity, μ = 10 mPa s (10-times water), density ρ = 1.125 g/cm^3 and interfacial tension with PP1 is 43.4 ± 0.1 mN/m.

A schematic of the experimental setup for the bubble or water droplets rise and collision observation is shown in fig. S1A. In essence, this is the same experimental setup that we used in our recent study on bubbles coalesce in PP11 (*19*). A glass container (cross section 2.5 × 2.5 cm, height 7.5 cm) is partly filled with the PP1 liquid. Usually we filled about 5 cm of the glass container height with the PP1 liquid, and added about 1 cm of water solution on top of it in the case of PP1-water solution interface experiments. Bubbles are released from the fine end of a glass capillary mounted close to the bottom of the container. The fine end of the bubble release capillary with inner diameter 2 μm to 5 μm was fabricated using a glass-puller. The other end of the capillary is connected by a plastic tube to a pressure regulator used to generate controlled air flow pulses. Using combinations of different capillary fine end diameters and pressure pulse duration we released air bubbles with diameters in the range of 50 μm to 1000 μm. To create water solution droplets in PP1, the fine-end capillary was connected by plastic tubing to a 10 ml syringe filled with the water solution, allowing the release of water droplets of diameters in the range of 50 μm to 1600 μm. Similar we could generate tetradecane droplets of diameters in the range of 100 μm to 900 μm.

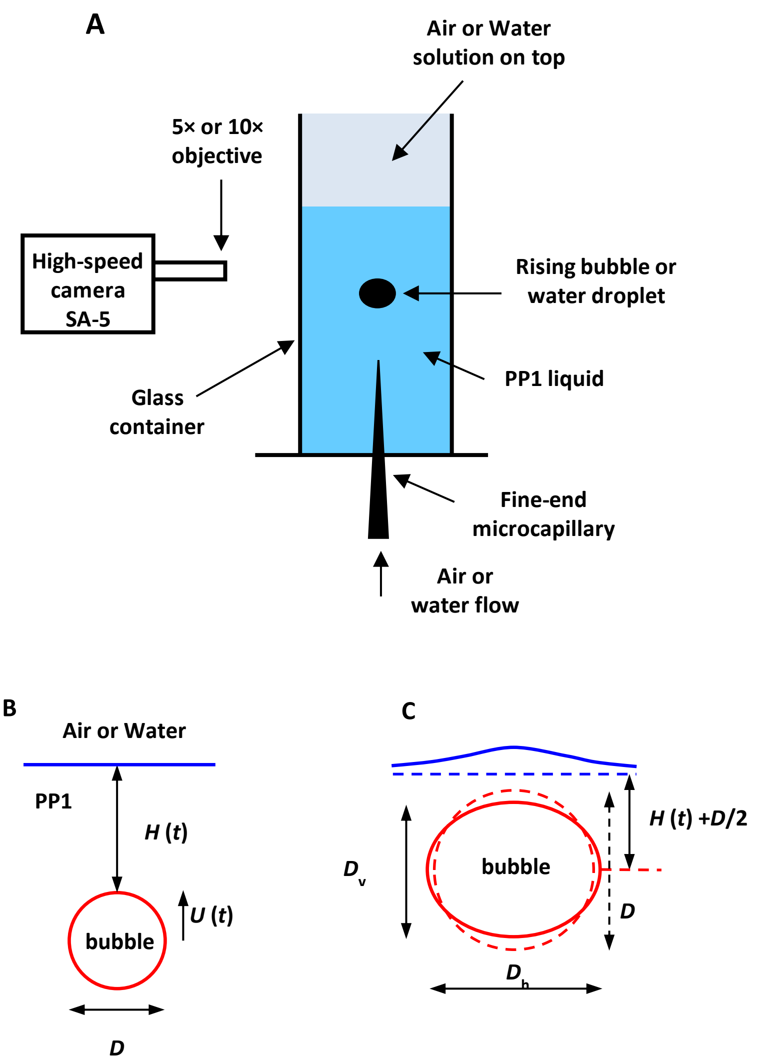

Fig. S1. Experimental setup and schematics. (A) Schematic of the experimental setup. **(B)** Schematic of a spherical bubble rising toward the flat PP1-air or PP1-water interface. **(C)** Schematic of an oblate ellipsoid bubble of horizontal diameter, D_h and vertical diameter, D_v approaching the deformable surface. Blue dashed lines denote the position of the undeformed interface and the undeformed bubble of dimeter $D \equiv (D_h^2 D_v)^{1/3}$ is indicated by the red dashed line. The bubble center-of-mass position, relative to the horizontal reference position is $H(t)$ as indicated, with $H(t) = 0$, corresponding to undeformed bubble in contact with undeformed interface. For undeformed bubble and interface, $H(t)$ is the same as the distance of the top of the bubble to the reference plane of the undeformed upper interface as shown in (B).

Section S3. Bubble and droplet terminal rise velocity

A simple and accurate way to evaluate the mobility of the bubble or droplet interface is to measure the terminal velocity, U_T of the free rising bubbles/droplets (*12*,*13*,*19*,*20*). In the case of small Reynolds number Re < 0.1, where the bubble or droplet remains spherical terminal velocity is given by Stokes result, U_{St} if the surface is immobile:

$$U_T = U_{St} \equiv (\rho - \rho_p)gD^2/(18\mu), \text{ (immobile)} \qquad \text{S1}$$

where ρ is the fluid density, ρ_p is the sphere density ($\rho_p \ll \rho$ for air bubble, $\rho_p \approx 1.0$ g/cm^3 for water droplet) and g is the gravitational acceleration. If the interface is mobile, the terminal velocity at small Re is given by the Hadamard-Rybczynsky result, U_{HR}:

$$U_T = U_{HR} \equiv [(\mu+\mu_p)/(2\mu+3\mu_p)]\ (\rho - \rho_p)gD^2/(6\mu), \text{ (mobile)} \qquad \text{S2}$$

that is larger than the Stokes' results by a factor (3/2) in the limit of a bubble with negligible viscosity, $\mu_p \ll \mu$. In the limit of a high viscosity drop, $\mu_p \gg \mu$, this gives the Stokes' law (S1), for a 'solid' sphere.

However due to the relevantly low viscosity of PP1 in our experiments even for the smallest bubbles $D \sim 50$ µm the Reynolds number Re > 0.1. In this case, one can use the expression for the drag coefficient, C_d by Mei et al. (*21*) for the terminal velocity of fully mobile spherical bubble valid for intermediate Reynolds numbers (1 < Re < 50):

$$C_d = (16/\mathrm{Re})\{1+[8/\mathrm{Re}+(1/2)(1+3.315\mathrm{Re}^{-1/2})]^{-1}\} \text{ (mobile)} \qquad \text{S3}$$

If the surface of the bubbles or droplet are immobile, the Schiller-Naumann (*23*) empirical relation for C_d is valid for 0.2 < Re < 1000:

$$C_d = (24/\mathrm{Re})(1 + 0.15\ \mathrm{Re}^{0.687}) \qquad \text{(immobile)} \qquad \text{S4}$$

Equations (S3) and (S4) give relations between the drag coefficients, C_d and the Reynolds number, Re = $\rho D U_T/\mu$. However, when the bubbles or droplet attains terminal velocity the buoyancy force, $F_B \equiv (\pi/6)(\rho - \rho_p)gD^3$ is balanced by the drag force, $F_D \equiv (\pi\rho D^2 U_T^2/8)C_d$, to give the implicit result for the terminal velocity: $U_T^2 = (4/3)[(\rho - \rho_p)/\rho]\ gD/C_d$, relating C_d with U_T and hence Re.

The Mei et al. and Schiller-Naumann results are for spherical bubble or droplets. The deformed shape of the bubbles or droplets can be approximated by an oblate ellipsoid with horizontal, D_h and vertical, D_v diameters (fig. S1C). The equivalent diameter, $D \equiv (D_h^2 D_v)^{1/3}$ provides a convenient measure of bubble size. The degree of deformation is measured by the aspect ratio: $\chi \equiv D_h/D_v$. For our system bubbles of size $D < 200$ µm, $1/\chi < 0.99$ and water solution droplets of $D < 500$ µm, $1/\chi < 0.98$, which justifies the applicability of (S3) and (S4) for that sizes range.

Experimental results for terminal velocities of small bubbles 50 µm < D < 200 µm and water solution droplets 50 µm < D < 500 µm are given in fig. S2, compared with the prediction of mobile (S3) and immobile theories (S4). In fig. S2A, the results are presented as terminal velocity, U_T *vs* bubble diameter, D, and in fig. S2B the same data are given as dimensionless terminal velocity, U_T/U_{St} *vs* the Reynolds number, Re. The bubble results are closely predicted by Mei et al. (*21*) values for mobile spheres and the water droplets results follow the immobile sphere correlation formula of Schiller-Naumann (*23*).

We also measure the rise velocity of small droplets of Millipore purified water without added surfactants. As shown in fig. S2, both droplets with surfactants and droplets of pure water without added surfactants gave identical terminal velocities over the range of droplet size investigated. It is well known that even trace amounts of impurities will immobilize the oil-water interface. In our pure water droplets experiments the small volume of water droplets can be easily contaminated as the water passes through the thin capillaries system used to generate the droplets. This result is consistent with our prior work on the rise velocity of water droplets in high viscosity PP11 (*19*).

For the larger bubbles sizes, investigated (D > 200 μm) bubble deformation is significant and its effects need to be taken into account. In this case, we compare the experimentally measured bubble rise velocities with prediction based on the Moore 1965 theory (*22*) for the drag on mobile deformable bubbles which is valid for the case of higher Reynold numbers, Re > 50. The following set of equations can be used to calculate the variation of the bubble terminal velocity with bubble size under these conditions (*20*):

The buoyancy force, F_B on the bubble is given by:

$$F_B = -\frac{1}{6}\pi D^3 \rho g \qquad \text{S5}$$

At the terminal bubble velocity the buoyancy force is balanced by the hydrodynamic drag force:

$$F_D = C_d Re \frac{\pi}{8} \mu D U_T \qquad \text{S6}$$

The product of the drag coefficient and the Reynolds number, C_dRe is given in terms of the aspect ratio χ according to the theory of Moore (*20*, *22*) as:

$$C_d Re = 48 G(\chi)\left(1 + \frac{K(\chi)}{\sqrt{Re}}\right) \qquad \text{S7}$$

$$G(\chi) = \frac{1}{3}\chi^{4/3}(\chi^2 - 1)^{3/2} \frac{\left[\sqrt{(\chi^2-1)} - (2-\chi^2)\sec^{-1}(\chi)\right]}{\left[\chi^2 \sec^{-1}(\chi) - \sqrt{(\chi^2-1)}\right]^2} \qquad \text{S8}$$

$$K(\chi) = 0.0195\chi^4 - 0.2134\chi^3 + 1.7026\chi^2 - 2.1461\chi - 1.5732 \qquad \text{S9}$$

And the relation between the aspect ratio χ and the Weber number, We = $D\rho U_T^2/\sigma$ is (*20*):

$$\frac{1}{\chi} = 1 - \frac{9}{64} We \qquad \text{S10}$$

Experimental results for the terminal velocity of deformed bubbles are compared with theoretical prediction (S5-S10) in fig. S3A, and the observed variation of bubble deformation with Weber number are compared with (S10) in fig. S3B. For reference we also include prediction based on the undeformed mobile bubble due to Mei at al. (*21*) and undeformed immobile sphere correlation due to Schiller-Naumann (*23*).

The results in Fig. 3 show excellent agreement with the mobile bubble theoretical predictions and confirm that for the entire range of bubble sizes investigated here the PP1-air interface behave as a stress-free mobile interface.

We notice that similarly to the case of bubble rise in PP11 liquid (*19*) the results in figs. S2 and S3 are very well reproducible and independent on the bubble surfaces prehistory. For example, there is no surface aging effect that is characteristic for water, where even exposure of the interface to the lab atmosphere can rapidly lead to immobilization of the interface due to the adsorption of trace contaminations (*12,13, 34*).

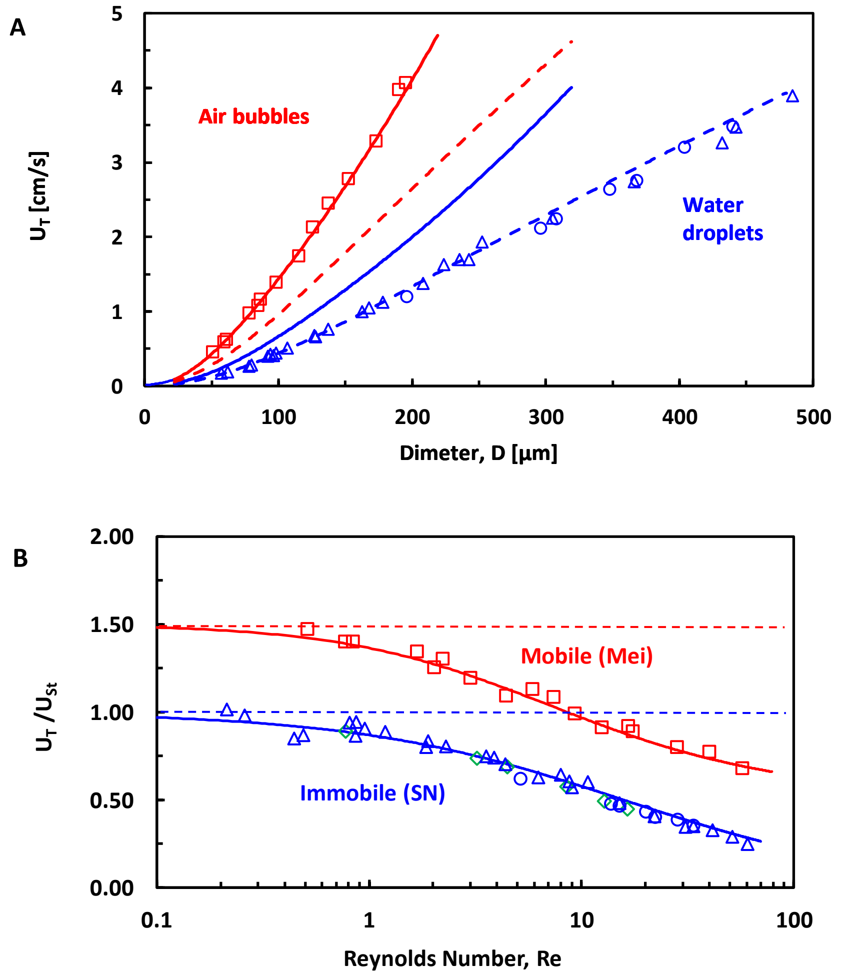

Fig. S2. Rise velocity for spherical bubbles and droplets. (A) Variation of the terminal rise velocity, U_T with the diameter of spherical bubbles (red square), spherical water surfactant solution droplets (blue triangles) or pure water droplets (open blue circles) in PP1. The solid lines are the theoretical prediction given by Mei et al., (S3) for mobile spherical bubbles (solid red line) or water droplets (solid blue line), and the dashed lines is the Schiller-Naumann correlation, (S4) for immobile spherical bubbles (dashed red line) or water droplets (blue dashed line). **(B)** The same data present as U_T/U_{St} *vs* Re. The solid red line is Mei et. al result for mobile bubble or droplet and the solid blue line is Schiller-Naumann (SN) dependence for immobile bubble or droplet. The green empty diamonds are for the rise velocity of tetradecane droplets in PP1.

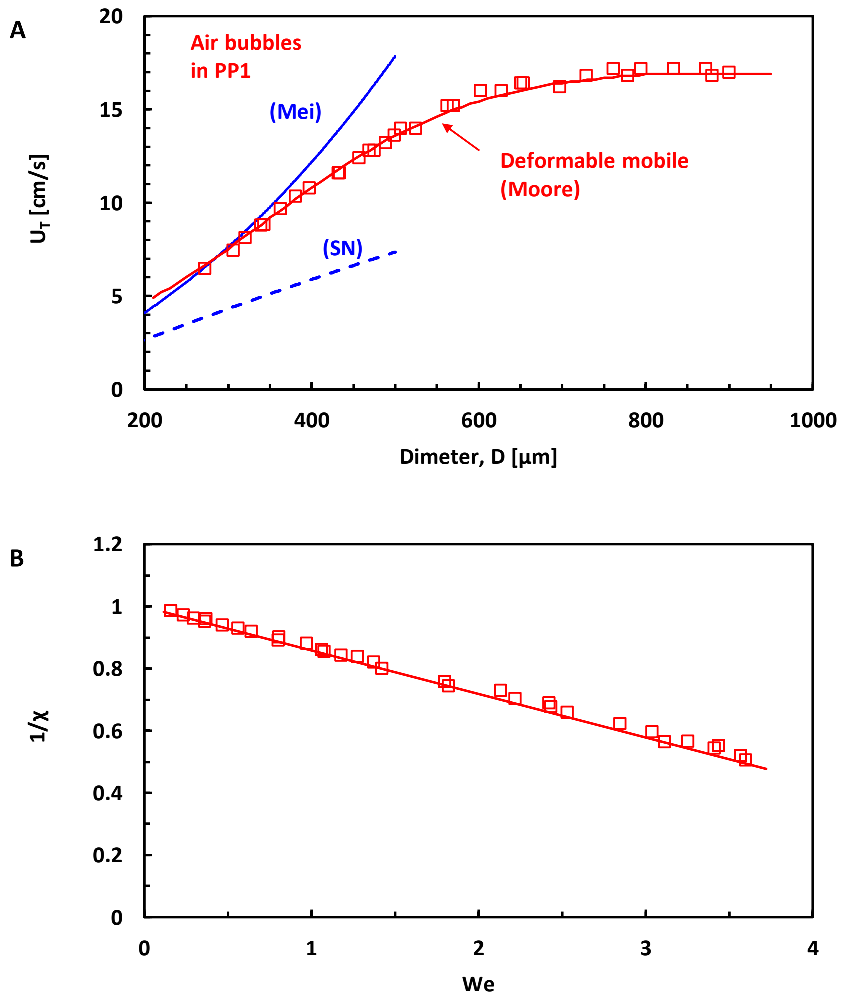

Fig. S3. Rise velocity for larger bubbles. (A) Dependence of terminal rise velocity, U_T on the bubble diameter for larger bubble sizes, $D > 300$ μm (Re > 50), experimental data (red squares) comparison with the Moore theory for mobile deformable bubbles (solid red line, (S5-S10)). Shown also are the Mei et al. result for spherical mobile bubbles (solid blue line, (S3)) and the Schiller-Naumann equation for immobile sphere (dashed blue line, (S4)). **(B)** Variation of the aspect ratio, $1/\chi$ of deformed bubbles with Weber number, We. Solid line is equation (S10) and red squares are the experimental data points.

Section S4. Drainage time experiments details

For our system the final outcome of the bubble or droplet collision with the interface is determined by the sign of the van der Waals interaction force (*24*). For bubbles colliding with the PP1-air interface, this force for the air-PP1-air film is attractive and will cause rapid coalescence. However, for bubbles colliding with the PP1-water interface this force is repulsive resulting in the formation of a stable air-PP1-water thin liquid film that keeps the bubble stable just below the interface. Similarly, for water droplets colliding with PP1-air interface, the water-PP1-air van der Waals force is repulsive and the droplet is held at below the interface. However, for water droplets at the PP1-water interface, or the van der Waals force for the water-PP1-water film is again attractive and the droplets eventually coalesce with the upper water phase.

In the cases when the bubble or droplet will coalesce with the upper phase, the time scale of the coalescence is determined by the drainage time of the thin liquid film separating them from the upper phase. In the case of bubble coalescing with the PP1-air interface, representing a mobile interfaces PP1 thin liquid film, following the bubble rebounds the final coalescence occurs almost instantaneous without apparent delay due to film drainage when the standard filming speed of 5000 frames per second (fps) is used (movie 1 (left)). To obtain a more accurate estimate for the final coalescence time scale we use higher filming speeds of up to 50,000 fps. As demonstrated in the snapshots sequence taken from such a video (Fig. 3A), even at these high filming speeds it was difficult to detect a static state of the bubble been held at the interface before coalescence. Thus we estimate that the characteristic drainage times for the bubble vs PP1-air coalescence is always bellow one millisecond (< 1 ms).

In contrast to the bubble vs PP1-air system, the water solution droplets stay for a considerable time (of order seconds) at the interface before coalescing with the upper water solution phase. However, the drainage times measured for water-PP1-water thin liquid films had poor reproducibility, ranging from 0.1 to 10 seconds between different experimental runs. The poor reproducibility of thin liquid films including water phase interfaces has been observed as well in prior experiments with higher viscosity PP11 liquid (*19*) and the most probable reason is the extreme sensitivity of the properties of the PP1-water interface to even small amounts of surface

active contaminations. In some experiments, we were added higher amount of electrolyte (0.5 M NaCl) to the water phase for both pure water and water solution with added Triton-X 100 in order to screen possible electric charges effect. However, this did not improved the reproducibility of the measured drainage times.

To examine the time scale for the drainage of a PP1 thin liquid film trapped between two immobile deformable interfaces, we conducted experiments using tetradecane oil droplets coalescing with P11-tetradecane interface. These experiments showed much better reproducibility compared to the water-PP1-water films experiments. Measurement of the terminal rise velocity of small tetradecane droplets confirmed that these droplets behave as immobile interface droplets (fig. S3B, green circles data points). The snapshots sequence in the manuscript Fig. 4B gives an example for the time scale of the film drainage time, with droplet been held on the interface for 2.5 seconds before coalescence. Drainage times for tetradecane droplets vs PP1-tetradecane (Fig. 4C) are more than three orders of magnitude longer than for the case of mobile interface bubble at the PP1-air interface.

In comparing the drainage time of the air-PP1-air films to water-PP1-water or tetradecane-PP1-tetradecane films we should note that apart from the interface mobility there are other factors that determine the life time of the films, these include interfacial tension, buoyancy, interfacial force as the magnitude of the van der Waals force, interface charge, etc. A more quantitative approach to estimate the surface mobility effects is to compare the measured drainage times with the prediction of theory model as we done before for the higher viscosity PP11 liquid thin films (*19*). Nevertheless, we assume that one primary factor for the order of magnitude difference is the life time of air-PP1-air films and water-PP1-water or tetradecane-PP1-tetradecane films is the interfaces mobility, with quantification of this effect to be conducted in future studies.

Section S5. Gerris DNS

To model the bubble and droplets collision with the interface we use the Gerris code (*26*-*31*) to solve the incompressible Navier-Stokes equations with the volume-of-fluid (VOF) method. The code uses adaptive mesh approach which makes it efficient in modeling problems related to bubble collision with an interface.

Bubble bounce from PP1-air, droplet bounce from PP1-water and bubble and droplets bouncing from the flat glass interface were simulated using the generic two-phase VOF Gerris code. To simulate the bubble bounce from the PP1-water interface and droplet bounce from the PP1-air interface we had to use three-phase VOF, following the approach given in Chen et al. (*30*). In Gerris the no-slip boundary condition can be defined only for a solid undeformed interface. Our approach to simulate the deformable immobile water droplets was to prescribe an effective higher viscosity of the water phase as detailed below.

Typical simulation domain used for the simulating the bubble/droplet rise toward the deformable interface or flat solid are shown in fig. S4A. In the beginning of the simulation the refinement level used for the adaptive mesh was kept to 11. Once the bubble or droplet collides with the interface we gradually increase the refinement level to avoid coalescence with the interface. In the case of water droplets a refinement of 11 to 14 was sufficient for bounce without coalescence. However, for the case of bubble bounce form the free PP1-inteface (movie 3) we used a maximum mesh refinement up to16 to simulate the bubble bounce from the interface.

The simulation of air bubble rise toward the free PP1-air interface or a solid surface was done using the same parameters as in the experiment. Air has density of 1.21 kg/m^3, and viscosity of 1.81 × 10^{-2} mP s. The PP1 has a density of 1718 kg/m^3 and a viscosity of 0.78 mPa s. The PP1-air surface tension was set to 12.4 mN/m. Gravity was accounted with g = 9.81 m/s^2. As shown on fig. S4B using the level 15 refinement resulted in minimum film thicknesses of about 160 nm. Excellent agreement with experiment is achieved as shown in Fig. 1E graph comparison.

The simulation of water solution droplet vs PP1-water solution was done using the same parameters as in the experiment except for the water viscosity. The water density of 997.8 kg/m^3 and water solution – PP1 interface tension of 12.4 mN/m was used. In fig. S5A we show the experimental center of the mass position vs. time for the 1080 μm water solution droplets bouncing from the PP1-water interface with simulation using the actual water viscosity (red line) and 10 times water viscosity (blue line). Because of interface mobility the simulation using the actual water viscosity (1.3 times the PP1 viscosity) over-predicts the droplet rise velocity and bounce amplitude. Using a viscosity that is 10 times higher for the water phase (about 13 times the PP1 viscosity) effectively immobilizes the interface to give reasonable agreement with the experiment. In other simulation we used effective viscosity as higher as 100 times the water viscosity. In this case the rise velocity of the water droplet was even closer to the experimental, however the bounce amplitude was significantly less than the experimentally observed, which is probably due to the effective stiffening of the water phase by the very high effective viscosity.

The simulation of the bubble bounce from the PP1-interface was done by introducing a third top phase which has water density and 10 times water viscosity to effectively impose the non-slip boundary condition. As show in fig. S5B similarly as in the case of water droplet vs PP1-air simulation using the actual water viscosity for the top phase (red line) give a larger bounce compared to the simulation using 10 time water viscosity for the top phase (blue line). Respectively the three-phase simulation of the water droplet bounce from the PP1-air interface was done using water density and 10 time water viscosity for the water droplet and air density and air viscosity for the top phase.

Due to symmetry considerations, the simulation of the head-on collision between two identical droplet or bubbles in liquid is equivalent with the simulation of the collision of a droplet or bubble with a free-slip solid wall which can be readily done using the Gerris code. To compare the collision of low and high mobility emulsion droplets we use the same approach as in the droplets collision with PP1-water interface simulation comparing water viscosity droplets collision (high surface mobility) with ten times higher viscosity water droplets collision (low surface mobility). To enhance the bouncing, we used higher surface tension droplets equal to that of the pure water – PP1 interface of 55.0 mN/m. The initial acceleration of the droplets was done

as in the free-rise bubble/droplets simulation using the gravity field. However, once the droplet reaches certain distance from the interface the ($2R$ for movie 9 example and $1R$ for movie 10) the gravity field is remover to simulate head-on collision without external forces. In the simulation results presented in movie 9 and 10 we used the same computational domain and droplets starting position as schematized in fig. S4. However, in this case we have imposed no-slip boundary condition on the side wall, which will correspond to the practical situation of a droplets in a cylindrical channel. We also conducted the same simulation using a much wider channel (15 droplets diameters instead of 6) with free-slip boundary reflecting collision of droplets in bulk liquid pool. Similar trends where observed as stronger bounce of high mobility droplets and droplets secondary collision.

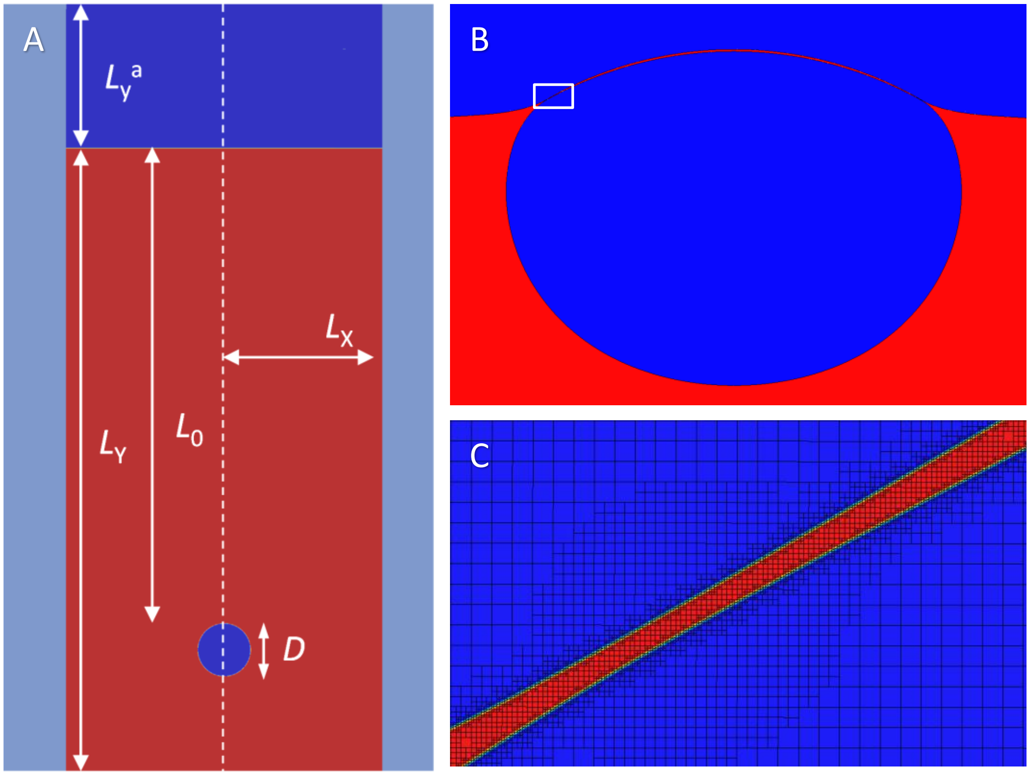

Fig. S4. Computational domain and adaptive mesh. (A) Initial condition in the Gerris simulation of bubble/droplet rise in PP1 and rebound from the interface. Red is PP1 liquid, blue is air or water, D = 1/3, L_x = 1, L_Y = 4, L_0 = 3, L_Y^a = 1. The domain of computation is limited to the left hand side, due to the axisymmetric flow condition. In the bubble bounce from the PP1-water interface the top domain is assigned water density and 10 times water viscosity and in the case of droplet bounce form the PP1-air interface the top domain is air. In the bubble bounce from a solid surface and droplets collision simulation the same liquid domain is used with the top boundary a solid (free-slip or no-slip) interface **(B)** Contour image of the bubble during the bounce form the interface about the moment of minimum film thickens **(C)** Zoomed-in portion indicated by the white rectangular area on (B) showing the adaptive mesh of level 15 refinement used in this case to resolve the PP1 thin liquid film of approximate thickness of 160 nm.

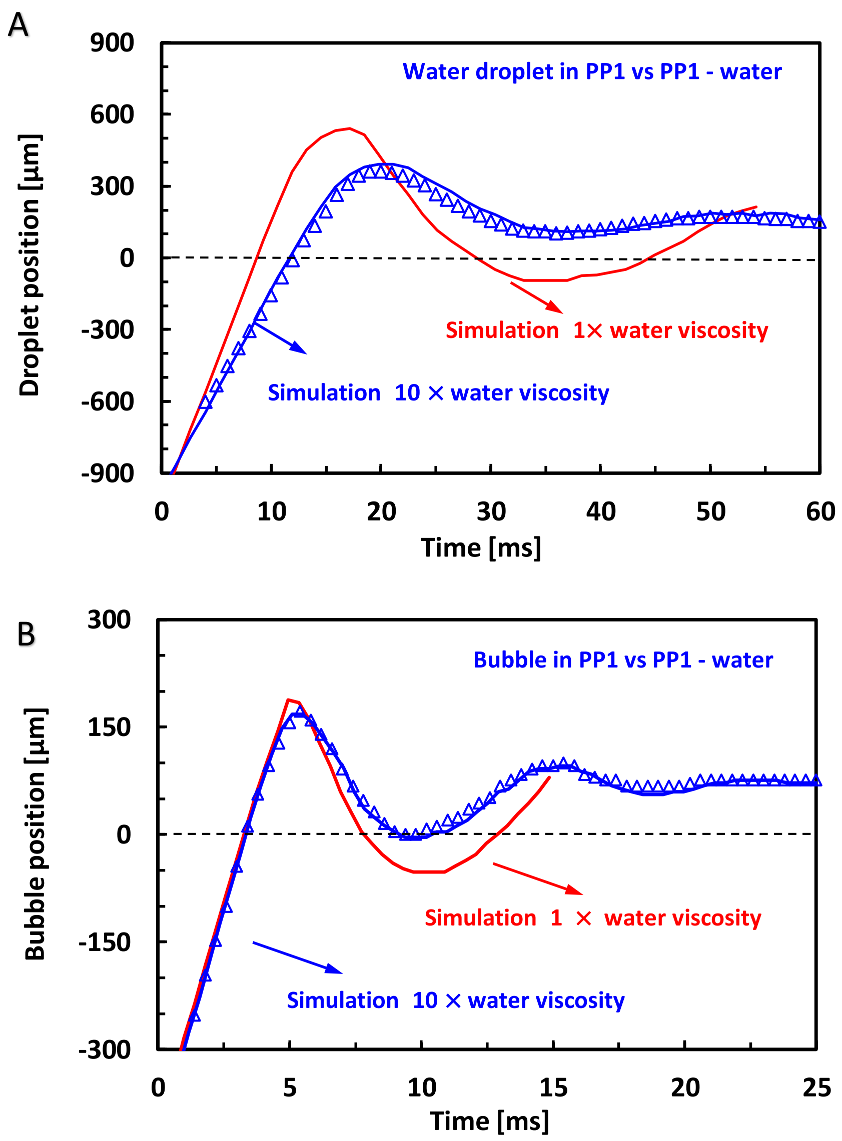

Fig. S5. Comparison between experiment and simulation. (A) Comparing of experiment center of the mass position (open triangles, blue) and DNS results using the actual water viscosity (red line) or 10 times water viscosity (blue line) for a 1080 µm water solution droplet bouncing from the PP1-water solution interface. **(B)** Comparing of experiment center of the mass position (open triangles, blue) for a 480 µm bubble bouncing from the PP1-water solution interface with DNS results using the actual water viscosity (red line) for the top phase or 10 times water viscosity (blue line).

Movies legends:

Movie S1. This combined movie shows the bouncing of a bubble of 480 µm undeformed diameter from the free PP1-air interface (left) or the PP1–water solution interface (right) of equal deformability. The movie is shot at 5000 fps and played at 30 fps.

Movie S2. This combined movie shows the bouncing of a 1080-µm water solution droplet from the PP1-air interface (left side) or the PP1–water solution interface (right side) of equal deformability. The movie is shot at 5000 fps and played at 30 fps.

Movie S3. This movie compares experiment (left) with simulation result (right) for the bouncing of a 480–µm–undeformed-diameter bubble from the free PP1-air interface. The simulation uses the same physical parameters for the PP1 liquid and bubble as the experiment. Time interval per frame is 0.2 ms (5000 fps) and movie is played at 30 fps.

Movie S4. This movie compares experiment (left) with simulation result (right) for the bouncing of a 480–µm–undeformed-diameter bubble from the PP1–water solution interface. The simulation uses the same physical parameters for the PP1 liquid, bubble and water solution as the experiment except for the water viscosity which is ten times higher than the water actual viscosity. Time interval per frame is 0.2 ms (5000 fps) and movie is played at 30 fps.

Movie S5. This movie compares experiment (left) with simulation (right) for the bouncing of a 1080–µm–undeformed-diameter water solution droplet from the PP1–water solution interface. The simulation uses the same physical parameters for the PP1 liquid, water droplet and air as in the experiment, except for the water viscosity which is ten times higher than the water actual viscosity. Time interval per frame is 0.4 ms (2500 fps) and movie is played at 30 fps.

Movie S6. This movie compares experiment (left) with simulation (right) for the bouncing of a 1080–µm–undeformed-diameter water solution droplet from the free PP1-air interface. The simulation uses the same physical parameters for the PP1 liquid, air phase and the water droplet as in the experiment, except for the water viscosity which is ten times higher than the water actual viscosity. Time interval per frame is 0.4 ms (2500 fps) and movie is played at 30 fps.

Movie S7. This movie compares experiment (left) for the bouncing of a bubble of 480 µm undeformed diameter free-rising in PP1 from a flat glass surface with simulation for the bubble bounce from no-slip solid flat (middle) or a free-slip solid flat (right). The simulations use the same physical parameters for PP1 and the air bubble as the experiment. Time interval per frame is 0.2 ms (5000 fps) and movie is played at 30 fps.

Movie S8. This movie compares experiment (left) for the bouncing of a water-glycerol mixture droplet of 1550 µm undeformed diameter free-rising in PP1 from a flat glass surface with simulation for the droplet bounce from no-slip solid flat (middle) or a free-slip solid flat (right). The simulations use the same physical parameters for PP1 and the water-

glycerol mixture droplet as the experiment. Time interval per frame is 0.6 ms and movie is played at 30 fps.

Movie S9. Simulation for the collision of the two pure water droplets of 1.2 mm undeformed diameter in PP1 for the case of low-mobility water droplets (10× water viscosity, the upper pair) or high-mobility water droplets (1× water viscosity, the lower pair). Initial droplets acceleration is done using gravity field, which is switched off at 4*R* droplets separation. The entire simulation is done using level 11 mesh refinement. Simulation time interval per frame is 0.51 ms and movie is played at 30 fps.

Movie S10. Simulation for the collision of the two pure water droplets of 1.2 mm undeformed diameter in PP1 for the case of low-mobility water droplets (10× water viscosity, the upper pair) or high-mobility water droplets (1× water viscosity, the lower pair). Initial droplets acceleration is done using gravity field, which is switched off at 2*R* droplets separation. The entire simulation is done using level 11 mesh refinement. Simulation time interval per frame is 0.51 ms and movie is played at 30 fps.

Movie S11. Velocity field visualization in the simulation of the collision of the two 1.2-mm pure water droplets in PP1 of low surface mobility with gravity switched off at 2*R* droplet separation (movie S10, top droplet pair case). For the horizontal velocity color scale legend see manuscript Fig. 6.
